# Orofacial Granulomatosis among Pediatric Patients Well Controlled by Corticosteroid Treatment: A Rare Case Series

**DOI:** 10.1155/2024/5685686

**Published:** 2024-04-29

**Authors:** Taku Kimura, Ken-Ichiro Sakata, Shunichiro Takezaki, Takuya Asaka, Shohei Oshima, Aya Yanagawa-Matsuda, Yoshimasa Kitagawa

**Affiliations:** ^1^Department of Oral Diagnosis and Medicine, Faculty of Dental Medicine, Hokkaido University, Sapporo 060-8648, Japan; ^2^Department of Pediatrics, Hokkaido University Hospital, Sapporo 060-8638, Japan; ^3^Department of Dentistry for Children and Disabled Person, Graduate School of Dental Medicine, Hokkaido University, Sapporo 060-8648, Japan; ^4^Department of Vascular Biology, Faculty and Graduate School of Dental Medicine, Hokkaido University, Sapporo 060-8586, Japan

## Abstract

Orofacial granulomatosis (OFG) is a rare disease entity characterized by nonnecrotizing granulomatous inflammation in the oral and maxillofacial regions, typically characterized by recurrent or persistent edema, primarily in the lips and occasionally in the gingiva. OFG is often associated with Crohn's disease and sarcoidosis, and an accurate diagnosis requires systemic examination of patients. Pediatric patients possess unique oral conditions where dental plaque rapidly forms, especially during tooth replacement due to tooth crowding. Moreover, controlling oral hygiene can be challenging, rendering it difficult to distinguish plaque-induced gingivitis from nonplaque-induced gingivitis. We elucidate the reports of pediatric patients who developed OFG in the lips and/or gingiva alone, which was well controlled through corticosteroid treatment. The patients demonstrated recurrent lips and/or gingival swelling with redness, which failed to improve despite oral health care and treatment with antibiotics and/or corticosteroid ointment. Incision biopsy was performed, which demonstrated granulomatous inflammation. Further systemic examination ruled out Crohn's disease and sarcoidosis and confirmed OFG diagnosis. Corticosteroid treatment orally or through gargling was administered to the patients, which provided improvement of symptoms after 1 month. As OFG may be associated with intractable diseases, monitoring the patient regularly is crucial. Pediatric patients with OFG require a collaborative approach with pediatricians and pediatric dentists to manage their oral and overall health.

## 1. Introduction

Orofacial granulomatosis (OFG) is a rare disease entity characterized by nonnecrotizing granulomatous inflammation in the oral and maxillofacial regions. Recurrent or persistent edema, particularly in the lips, oral ulcers, and gingivitis are among the primary manifestations of OFG. OFG is often correlated with systemic diseases such as Crohn's disease and sarcoidosis. When OFG is only present in the lips, it is referred to as granulomatous cheilitis (GC). If OFG presents with unilateral facial nerve palsy and a fissured tongue, a diagnosis of Melkersson–Rosenthal syndrome is established. Thus, distinguishing OFG from other systemic diseases is pivotal. The etiology of this disease has yet to be elucidated; however, it is reported to be associated with allergies and related diseases such as hay fever, bronchial asthma, and atopic dermatitis [1–3]. Furthermore, OFG tends to develop in individuals aged 20–40 years and limited literature has described its occurrence and management in pediatric patients [4, 5]. Herein, we present a series of four pediatric patients who developed OFG in the lips and gingiva in one case and gingiva alone in three cases. Each case was successfully managed with corticosteroid treatment.

## 2. Case Presentation

**Figure 1:**
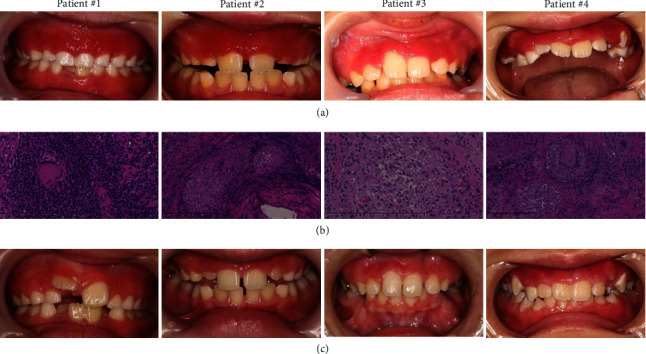
Clinical and pathological characteristics of four pediatric patients with orofacial granulomatosis. (a) Each showed bleeding edema with upper anterior gingival redness. (b) Incision biopsy demonstrated noncaseating granuloma with infiltrating lymphocytes, plasmacytes, epithelioid histocytes, and multinucleated giant cells beneath the mucosal epithelium (patient #1 and 3: magnification 400x; #2 and 4: magnification 200x). (c) Local or systemic corticosteroid treatment improved oral manifestation.

The four patients diagnosed with OFG are summarized in Table 1 and Figure 1.

Their ages ranged from 7 to 9 years. Patients #1 and #2 had allergies to household dust and tree nuts, respectively, and patient #2 had a history of bronchial asthma. All patients had recurrent upper gingival swelling and bleeding in the region when brushing, and patient #1 also presented with recurrent labial swelling. Prior to referral to the Oral Diagnosis and Medicine Department at Hokkaido University Hospital, patients underwent antimicrobial therapy with amoxicillin or cefcapene pivoxil and corticosteroid ointment application, which failed to improve their symptoms.

The four patients had poor oral hygiene and presented with edema with redness in the upper anterior gingiva. Panoramic X-ray and/or computed tomography scans showed no signs of bone resorption adjacent to the lesion in each patient. Blood tests revealed that patients #2 and #3 had high IgE levels. Thus, the initial diagnosis for patient #1 was plaque-induced gingivitis, whereas patients #2 and #3 were diagnosed with allergic gingivitis. In addition, patient #4 was diagnosed with plaque-induced gingivitis and suspected hereditary angioedema (HAE).

First, all patients received oral health care to improve their oral hygiene in collaboration with the Pediatric Dentistry Department at Hokkaido University Hospital. These patients received tooth brushing instructions and professional mechanical tooth cleaning (PMTC) by pediatric dentists. However, these patients demonstrated persistent swelling in the upper gingiva 1 month after the therapeutic intervention, ultimately excluding plaque-induced gingivitis.

Furthermore, all patients underwent incision biopsy from the edematous regions under local anesthesia, which revealed granulomatous inflammation. To evaluate whether the oral manifestation was an associated symptom of systemic disease, pediatric consultation with the Department of Pediatrics at Hokkaido University was obtained for all patients. Eye and cardiac examination, fecal occult blood test, and chest radiography were performed, and no patient presented with any signs of Crohn's disease or sarcoidosis. A further blood test was conducted on patient #4, who developed recurrent labial swelling. The test demonstrated normal serum C4 levels and no signs of C1 inhibitor deficiency, which resulted in the exclusion of HAE. Patient #1 received local corticosteroid treatment with gargling 0.01% dexamethasone solution after each meal, whereas patients #2, 3, and 4 received systemic corticosteroid treatment orally, which successfully improved their symptoms. The corticosteroid dosage was gradually reduced, and patients #2 and #4 showed mild to moderate side effects, including declining adrenal cortex function, moon faces, and weight gain. Signs and symptoms improved after both patients completed corticosteroid treatment. Conversely, patient #3 experienced recurrence of upper gingival swelling 5 months after completing the corticosteroid treatment. Since the patient #3 presented mild swelling without any pain and did not exhibit further symptoms associated with systemic diseases, the patient did not receive any treatment. All patients are currently being monitored regularly.

## 3. Discussion

We herein present the reports of pediatric patients who developed OFG in the lips and/or gingiva alone, which could be controlled through corticosteroid treatment. In up to 70% of the cases, OFG typically occurs in the lip area and rarely in the gingiva. OFG limited to the lips is also called GC, which sometimes develops concurrently with Crohn's disease [6]. Approximately 20%–50% of Crohn's disease cases present with OFG, and oral OFG lesions precede gastrointestinal lesions in 5%–10% of the patients [7–10]. Patients with sarcoidosis, a chronic granulomatous inflammation affecting multiple organs in the body, also develop OFG, which presents as a submucosal mass or oral ulcer in approximately 3% of the patients [10–12]. Furthermore, oral manifestations precede sarcoidosis in 30–60% of the cases [10, 13]. Although Crohn's disease and sarcoidosis were ruled out in our patients according to systemic examinations, they still can develop these diseases. Thus, patients with OFG, especially pediatric patients who rarely complain of symptoms, should have their systemic condition monitored regularly for early disease detection. In addition, recurrent and/or persistent labial swelling in pediatric patients should be differentiated from autosomal dominant C1 esterase inhibitor deficiency, HAE. HAE typically develops in individuals under 20 years of age and those with a family history of similar orofacial swelling. HAE can be ruled out through the evaluation of complement protein levels in the blood, including C1-inhibitor and C4 [14], and should be considered in pediatric patients with recurrent labial and/or gingival swelling. In this study, patient #4 developed recurrent labial and gingival swelling, and blood tests aided in excluding HAE.

Gingival OFG presents similar features to marginal and eruption gingivitis in pediatric patients. Generally, pediatric patients have poor oral hygiene because of their unique oral condition, where dental plaque forms easily, especially during the teeth replacement period [15]. At their first appearance in our department, our patients had poor oral hygiene. We first provided oral health care with tooth brushing instructions and PMTC and prescribed antibiotics to relieve inflammation; however, gingival swelling and redness persisted. During this time, each patient's oral hygiene improved, which allowed us to rule out plaque-induced gingivitis. Distinguishing OFG from other diseases according to the clinical course and oral manifestations alone is difficult. However, a combination of clinical observations with pathological findings is crucial for an accurate OFG diagnosis. OFG presents with noncaseating granulomatous inflammation composed of lymphocytes, epithelioid histocytes, and multinucleated giant cells [16]. After a granulomatous inflammation diagnosis, further systemic examinations should be conducted to rule out Crohn's disease and sarcoidosis. In this study, all patients underwent incision biopsy during the early period when they presented to our department, and incision biopsy revealed typical pathological findings of OFG, which helped us refer the patients to the Pediatrics Department (Figure 1). Thus, incision biopsy is recommended for patients with gingivitis that is poorly controlled by oral hygiene.

The etiology of OFG remains to be elucidated; however, allergy is suspected to be a contributing factor [3, 17–19]. Studies reveal that people with a history of allergic diseases such as hay fever, bronchial asthma, and atopic dermatitis are more likely to develop OFG (12–60%) than those without such a history (up to 15%) [10, 20, 21]. In our cases, two of the four patients had allergy or allergic diseases; however, both had a favorable prognosis after treatment. Research also suggests that contact sensitivity to food additives, including cinnamon, cacao powder, and benzoate, may be associated with the onset of OFG [17, 21–23]. Interestingly, 32% of patients with allergic cheilitis due to cinnamon powder showed clinical features and pathological findings similar to those of patients with OFG [17]. Moreover, OFG may develop from delayed hypersensitivity to dental materials such as amalgam, which contains inorganic mercury [21, 24]. Although our patients had no history of dental restorations, an allergy test using IgE evaluation was conducted, which limits our ability to conclude the possibility of an allergic background in OFG development. Thus, a thorough medical interview on allergy and dietary habits and comprehensive allergy tests are crucial for patients with intractable gingivitis.

The treatment of OFG typically involves local and/or systemic corticosteroids [5, 16, 25]. All our patients received corticosteroid treatment, resulting in a significant improvement in oral manifestations 1 month after treatment initiation. For the recurrent case, patient #3 exhibited mild oral manifestation and lack of apparent systemic symptoms, the patient did not receive any treatment. However, considering the potential risks of developing the systemic diseases, patients with recurrent OFG should be performed systemic evaluation depending on the severity of oral manifestations and recurrent frequency. In addition, further corticosteroid treatment should be considered for treating patients with recurrent OFG. Corticosteroid treatment is a promising strategy for OFG treatment; however, side effects may occur in high-dose and/or long-term treatment. Long-term corticosteroid treatment can increase osteoporosis risk, which can induce growth suppression, particularly in pediatric patients [26]. Based on our patients' clinical course that patient #1 was well controlled by local corticosteroid treatment with gargle, local corticosteroid treatment with gargling as an initial treatment should be considered to prevent them from suffering systemic side effects. Recently, nonsteroidal therapy, long-term azithromycin treatment, TNF-*α* modulators, and sulfonamides are reported to be alternative treatment options, each based on therapeutic experiences in other granulomatous diseases [27–29]. However, evidence for these treatment options for patients with OFG has not yet been established. Randomized control trials employing patients with OFG according to etiology are warranted to establish treatment efficacy.

OFG can hamper patients' oral function and sometimes present as part of intractable systemic diseases such as Crohn's disease and sarcoidosis. Thus, systemic examination should be considered for pediatric patients who present with recurrent labial and/or gingival swelling that cannot be improved through oral health care. In addition, early incision biopsy can aid in OFG diagnosis. Regular long-term monitoring is crucial for patients with OFG, considering the possibility of recurrence and development of potential systemic diseases. Collaboration between pediatricians and pediatric dentists is vital to manage the systemic and oral health of these patients.

## Figures and Tables

**Table 1:** Clinical characteristics of four pediatric patients with orofacial granulomatosis.

Characteristics		Patient #1	Patient #2	Patient #3	Patient #4
Background	Age (years)	Seven	Seven	Nine	Seven
	Sex	F	F	M	M
	Medical histories	Allergy to household dust	Allergy to tree nuts, bronchial asthma, and constipation	ADHD	N/A
	Complaints	Recurrent upper gingival swelling and bleeding while brushing			Recurrent labial and upper gingival swelling and bleeding while brushing
	History of therapeutic intervention	Local corticosteroid treatment antimicrobial treatment with cefcapene pivoxil	Local corticosteroid treatment	Antimicrobial treatment with cefcapene pivoxil	Antimicrobial treatment with amoxicillin and antiallergic treatment with bepotastine, a selective antagonist of the histamine 1 receptor

First diagnosis		Plaque-induced gingivitis	Allergic gingivitis		Plaque-induced gingivitis and suspicion of HAE

Blood test	WBC (×10^4^/*μ*L)	9	4.4	5.7	7.3
	Platelets (×10^4^/*μ*L)	308	289	351	367
	C-reactive protein (mg/dL)	<0.02	0.22	<0.02	<0.02
	IgE (IU/mL)	N/A	1055.4	7566.9	N/A
	C1 inactivator (%)	119	N/A	N/A	N/A
	C4 (mg/dL)	22	38	27	19
	CH50 (U/mL)	55.6	60.3	52	55.7
	sIL-2R (U/mL)	372	448	374	676
	KL-6 (U/mL)	210	368	241	478

Pathological examinations		Granulomatous inflammation			

Treatment strategies		Oral health care			
		Local corticosteroid treatment with gargle	Systemic corticosteroid treatment		

Side effects		None	Moon face, weight gain	None	Declining adrenal cortex function

Outcomes		No evidence of recurrence		Recurrence five month after the completion of corticosteroid treatment	No evidence of recurrence

## Data Availability

The datasets used and/or analyzed during the current study are available from the corresponding author on reasonable request.
